# Prevalence of anemia and associated factors in children living in urban and rural settings from Bata District, Equatorial Guinea, 2013

**DOI:** 10.1371/journal.pone.0176613

**Published:** 2017-05-03

**Authors:** Policarpo Ncogo, Maria Romay-Barja, Agustin Benito, Pilar Aparicio, Gloria Nseng, Pedro Berzosa, Maria A. Santana-Morales, Matilde Riloha, Basilio Valladares, Zaida Herrador

**Affiliations:** 1 Centro de Referencia de Control de Endemias (CRCE), Malabo, Equatorial Guinea; 2 National Centre of Tropical Medicine, Instituto de Salud Carlos III (ISCIII), Madrid, Spain; 3 The Spanish Tropical Diseases Research Network (RICET in Spanish), Madrid, Spain; 4 Ministry of Health and Social Welfare, Malabo, Equatorial Guinea; 5 Instituto Universitario de Enfermedades Tropicales y Salud Pública de Canarias, Universidad de la Laguna, La Laguna, Canary Islands, Spain; Universidade de Sao Paulo, BRAZIL

## Abstract

Anemia in children under 5 years of age is a global public health problem. According to the World Health Organization the current rate of anemia among preschool aged children in Equatorial Guinea is 66%. No information is available above this age. The cross-sectional Prevamal Survey was conducted in 2013 aimed at providing baseline data on malaria prevalence in children aged 2 months-15 years old. Sampling was carried out with the use of a multistage, stratified cluster strategy in the district of Bata, Equatorial Guinea. The χ2 test and adjusted Poisson regression models were applied to assess the association between social-demographic and economic factors, malaria and anemia. A total of 1436 children were tested, out of which 1,421 children (99%) were tested for anemia. Over 85% were anemic; out of them, 284 (24%), 815 (67%) and 111 (9%) children had mild, moderate and severe anemia, respectively. Severe anemia was more frequent among children aged 2–12 months old and those living in rural sites. About 47% tested positive for malaria via a rapid diagnostic test (RDT). This rate was significantly higher in rural villages (66%; p<0.001). The prevalence of anemia and malaria was higher in rural settings (p<0.001). On the other hand, anemia in urban areas displayed a heterogeneity and complexity that differed from the rural environment: in urban neighbourhoods, children with concomitant malaria infection were more likely to be anemic (adjusted prevalence rate (aPR):1.19; CI 95%: 1.12–1.28). Moreover, the prevalence of anemia was higher in children aged above 13 months compared to younger children (p<0.005). Belonging to the poorest wealth tertile were positively (aPR: 1.14, 95% CI: 1.05–1.24) and children’ parents being employees (aPR: 0.86, 95% CI: 0.76–0.96) or self-employed (aPR: 0.86, 95% CI: 0.76–0.97) *vs*. working in agriculture and/or fishing negatively associated with anemia among urban children. This marked urban-rural variation indicates the importance of targeting specific areas or districts. Strategies aimed at reducing malaria are clearly paramount in this country. Prevention and treatment of other factors associated with the etiology of anemia (e.g., iron deficiency) are also likely necessary to combat the burden of anemia in Equatorial Guinea.

## Background

Anemia remains one of the most difficult public health problems to manage in malaria-endemic countries of Africa [1], affecting more than half of children less than five years old [2]. It poses a major public health issue leading to an increased risk of child mortality. Furthermore, anemia has negative consequences on cognitive development and physical growth of children from infancy through to adolescence [1]. It also damages immune mechanisms, and is associated with increased morbidity rates [3]. However, due to the insidious nature of its presentation, mild-to-moderate degrees of anemia frequently remain undetected and untreated by health care workers [4].

It is estimated that nearly half of all cases of anemia are due to iron deficiency, while other causes of anemia are multifactorial and include parasitic infections and nutritional deficiencies [5]. Anemia and malaria are key health problems in pediatric populations in sub-Saharan Africa [6]. Malaria causes anemia through hemolysis and increased splenic clearance of red blood cells and cytokine-induced dyserythropoeisis [7]. A single episode of malaria or repeated episodes due to reinfection or failure to adequately clear parasitemia may result in life-threatening anemia, and, if untreated, death [8].

Pediatric malaria associated anemia represents a major challenge for public health in sub-Saharan Africa [9]. Actually, severe anemia probably accounts for more than half of all childhood deaths from malaria in Africa [10]. In continental Equatorial Guinea (EG), malaria is an holo-endemic disease and exhibits a year-round transmission pattern [11]. Data from the 2011 Health Survey (EDSGE-I) shown RDT-based malaria prevalence of 58.7% in children aged 6–59 months old living in the mainland [12]. Prevalence estimates by the World Health Organization (WHO) indicate anemia is present in 66% of preschool aged children in EG [13]. More recent data are missing for malaria and anemia. Moreover, data in childhood malaria and anemia above this age group are quite scarce. Therefore, the present study aims at describing the current prevalence of anemia and malaria, and their related factors, among children aged 2 months to 15 years old in the District of Bata, in Equatorial Guinea.

## Methods

### Study area and population

The target population was children aged 2 months to 15 years old living in Bata district, Equatorial Guinea. EG is located on the Gulf of Guinea and has a total area of 28,068 km^2^. The EG mainland geographical region covers 26,017 km^2^ and it is bordered by Cameroon on the north and Gabon on the south and east. The mainland region comprises four provinces: Centro Sur, Kie-Ntem, Litoral and Wele-Nzas, and has a population of about 315,625, belonging to four main ethnic groups: Fang, Bubi, Combe and Annobonese. Each province is divided into several districts. According to the latest national census (DHS, 2011), the district of Bata, with a population of 244,264 inhabitants, is the largest district in the country [14]. Bata district’s public health facilities comprise a network of ten health centers.

Malaria is hyperendemic in the country [15], and there are high incidence rates of many infectious diseases, such as tuberculosis and enteric infections caused by intestinal parasitoses [16,17]. The Equatorial Guinea Malaria Control Initiative (EGMCI) was introduced in the mainland region in 2007. The strategy consisted mainly in indoor residual spraying in Litoral and Kie-Ntem provinces, and mass distribution of long-lasting insecticide treated nets in Centro Sur and Wele-Nzas provinces. Case management was also improved in all four provinces, through the distribution of free artemisinin-based combination therapy [15]. The EGMCI initiative was implemented by the government of EG, in collaboration with several international organizations. With the withdrawal of external funding in 2011, the EGMCI ceased its malaria control activities in EG. This has reverted to first line treatment stock shortages since 2012 (EG Ministry of Health unpublished report).

### Study design

This cross–sectional study was carried out during June–August 2013 in the Bata district. It was part of a project called “Prevamal”, which aimed at providing baseline data on malaria prevalence, molecular characterization of *Plasmodium* and malaria vectors in the study area and information on malaria knowledge, practices and attitudes among the targeted population.

Multistage, stratified cluster sampling strategy was used. Primary sampling units were rural villages and urban neighbourhoods, which were randomly selected with probability proportional to size to improve accuracy in sample design. Secondary sampling units were randomly selected houses. Sample size was calculated using an expected malaria prevalence of 50%. The initial sample size was increased in prevision of missing data. All children aged 2 months-15 years old living in the selected households and not receiving malaria treatment at the moment of the survey were included. Other methodological aspects have been published elsewhere [18–20].

### Data collection

A pre-piloted questionnaire was administered to the household caretaker/head of the selected children by trained medical personnel. Data collection teams were supervised by staff from the Health Institute Carlos III (ISCIII, Spain) and the Ministry of Health and Social Welfare of Equatorial Guinea (MINSABS).

The questionnaire comprised closed ended questions. It was designed to gather information on socio-demographic characteristics, health status and history of previous episodes of malaria, malaria-related behaviours and household characteristics. The questionnaire was previously translated into the main local language, Fang, and the option to be interviewed in Spanish or Fang, both official languages in the country, was given to the care provider.

Hemoglobin (Hb) levels for children were ascertained using HemoCue (Ängelholm, Sweden). WHO hemoglobin levels were used as cut-off values to classify anemia [18]. The WHO reference cut-off values to diagnose anemia in children are as follows: children 6–59 months of age 109 g/l or less, children 5–11 years of age 114 g/l or less and children 12–14 years of age 119 g/l or less. Modifications based on altitude were not carried out, as Bata district is located at sea level. The anemic children received iron supplementation. Children with Hb level below 8 g/dL were referred to a nearby health facility. NADAL rapid diagnostic test (Nal von Minden, Moers, Germany) for malaria infection was performed in situ. This test is capable of detecting both *Plasmodium falciparum* and other *Plasmodium* species. Participants with positive rapid tests were immediately offered treatment according to the EG National Guidelines for the treatment of malaria.

### Statistical analysis

The collected data were double entered into a data entry file using EpiData software, V.3.1. Data were then transferred to STATA for windows version 14 (Stata Corporation, College Station) for data management and analysed according to the study objectives.

Two response variables were used: a binary one, indicating whether or not a person has anemia, and a response variable with four categories (for assessing factors related to anemia severity). The independent covariates comprised individual variables, that included sex, age, setting, RDT result, fever in the last week, ethnic group, use of bed net, and household variables: caregiver´s level of education, family source of income and the wealth index. Data on household assets (e.g., source of water, house construction materials, television, radio) were collected and used to create a wealth index by principal component analysis, and standardized against a normal distribution with a mean of zero and a standard deviation of one. All households in the total sample were divided into tertiles based on their asset index score.

Frequencies and percentages were used to summarize data and to explore the differences by socio-demographic variables. To assess factors related to anemia, χ2 test for categorical variables was performed.

In the present study the prevalence of anemia in most of the exposure categories was higher than 10%. As highlighted by Barros et al., the odds ratios (OR) are a good approximation of the true prevalence ratios (PR) only when the frequency of disease in the general population during the study period is low, otherwise the OR would exaggerate the true relative risks [21]. In this situation, binomial regression has been recommended for the estimation of RRs (and PRs) in multiple analysis. In our study, this statistical method could not estimate RR because convergence problems, therefore Poisson regression, which has also been recommended as a suitable method for estimating RRs, was used.

Crude Prevalence Ratios between potential risk factors and outcomes were first calculated by using Poisson regression. Adjusted Poisson regression models were then developed to assess the association between social-demographic and economic factors, and anemia. Comparisons for which p–values were below 0.05 were considered significant. All variables associated with each of the outcomes at the p <0.10 level were included in the multiple analysis. Bivariate and multiple analyses were also performed with stratification by setting, as many significant differences between rural and urban sites were found during the descriptive analysis.

### Ethics clearance

The study was approved by the ethical review board of the Health Institute Carlos III (CEI PI 22_2013-v3) and the Equatoguinean Ministry of Health (MINSABS in Spanish). Support letters were obtained from the MINSABS and the Hospital of Bata. The village and neighbourhood representatives were informed by an official letter from the MINSABS of the day of the visit and the scope of the study. Written informed consent was obtained from all patients prior to study inclusion. Written consent to publish from the legal parent or guardian for children to report the data were also obtained. Anonymity was assured. A written statement was also included on the introductory part of the questionnaires in which further information concerning the purpose of the study and the confidentiality of the research information was given. Data were analysed in anonymous form.

## Results

### Prevalence of anemia and related factors

A total of 26 urban neighbourhoods and 19 rural villages were sampled in Bata district. Sampled clusters contained 430 households. This resulted in a study population of 1,436 children, out of which 1,421 (99%) were tested for anemia. All subsequent analyses were based on these children with Hb measures.

Overall, 1,210 (85.2%) children had anemia. Of these, 284 (23.5%), 815 (67.3%) and 111 (9.2%) children had mild, moderate and severe anemia, respectively. Differences by sex were not statistically significant: 85.7% of males and 85.1% of females had anemia. Males aged 59 months or less had anemia more frequently than those boys above this age (p = 0.007), while no significant differences by age group were observed for females (Table 1).

**Table 1:** Anemia prevalence by age group and sex in children, Bata district, June-August 2013.

Age group	Total		Males		Females	
	n (%)	p value	n (%)	p value	n (%)	p value
<= 12 months	350 (87.72)	0.132	182 (89.22)	0.007	168 (86.15)	0.652
13–59 months	441 (85.63)		242 (88)		200 (83.33)	
≥ 5 years	419 (82.64)		180 (79.64)		241 (85.77)	

**Fig 1:**
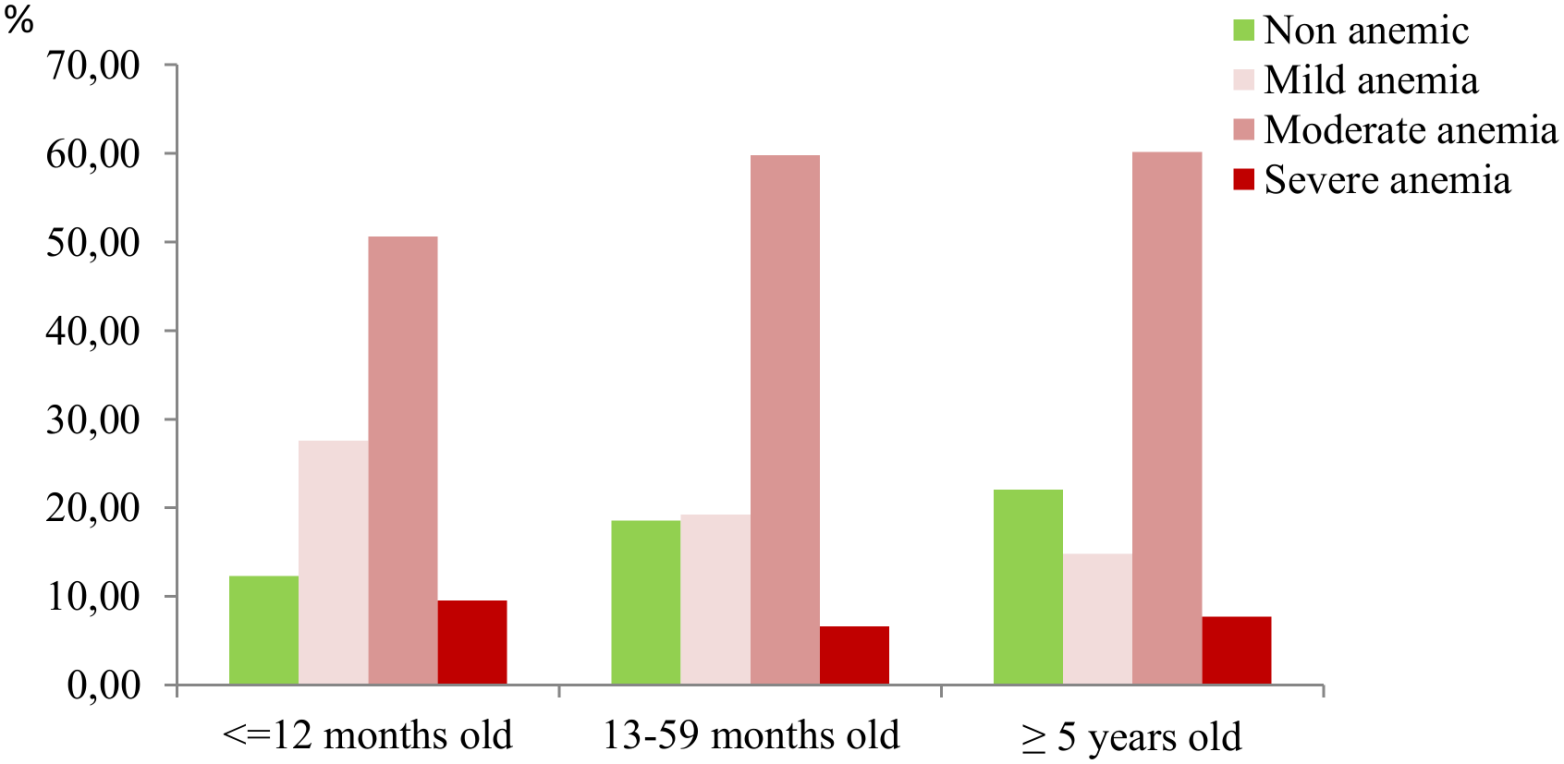
Percentage of anemia severity by age group in children, Bata district, June-August 2013.

By age group, prevalence of both mild and severe anemia was higher in those children aged 12 months old or less, while moderate anemia was more common among children aged 13 months–59 months old and over 5 years old (p < 0.001). Severe anemia was also less frequent in children aged 13 months–59 months old than those aged over 5 years old (Fig 1, S1 Table).

There were several differences by anemia severity and children´ socio-demographic and clinical characteristics. The percentage of children without anemia was higher in urban than in rural settings (17.3% and 10.3%, respectively), while severe anemia was more frequent among those children living in rural sites (p<0.001). Anemia severity and the caregiver´s educational level were also significantly associated: the percentages of children with moderate and severe anemia were higher among those children whose caregiver had less educational level (p = 0.021). Regarding the family incomes´ source, parents of most children without anemia or mild anemia were employees, while moderate and severe anemia were more frequent among children with household main incomes´ source being agriculture, fishing and/or chasing (p = 0.001). Children who belonged to the poorest wealth tertile had moderate and severe anemia significantly more commonly than those belonging to the richest tertile (p<0.001). The use of mosquito net was also significantly associated with a minor percentage of the most severe presentations of anemia, while having fever was associated with higher percentages of the most severe forms of anemia (p<0.001) (Table 2).

**Table 2:** Socio- demographic and clinical characteristics of the study population by anemia severity, Bata district, June-August 2013.

Variables		Anemia severity				p value
		Without anemia	Mild anemia	Moderate anemia	Severe anemia	
		n (%)	n (%)	n (%)	n (%)	
Sex	Female	108 (51.2)	149 (52.5)	406 (49.8)	53 (47.7)	0.811
	Male	103 (48.8)	135 (47.5)	409 (50.2)	58 (52.3)	
Setting	Rural	57 (27)	86 (30.3)	337 (41.3)	62 (55.9)	<0.001
	Urban	154 (73)	198 (69.7)	478 (58.7)	49 (44.1)	
Ethnic group	Fang	172 (81.5)	227 (79.9)	687 (84.3)	93 (83.8)	0.127
	Combe	12 (5.7)	22 (7.7)	65 (8)	6 (5.4)	
	Others	27 (12.8)	35 (12.3)	63 (7.7)	12 (10.8)	
Caregiver´s educational level	No formal education/Incomplete primary school	43 (22.1)	62 (25.3)	221 (29.5)	32 (33)	0.021
	Primary school	132 (67.7)	160 (65.3)	490 (65.3)	62 (63.9)	
	Secondary school	20 (10.3)	23 (9.4)	39 (5.2)	3 (3.1)	
Family incomes´source	Agriculture/Fishing/Chasing	10 (5)	16 (6.1)	106 (13.6)	12 (11.9)	0.001
	Employee	117 (58.2)	144 (54.8)	386 (49.6)	46 (45.5)	
	Self-employed	71 (35.3)	96 (36.5)	256 (32.9)	41 (40.6)	
	Others	3 (1.5)	7 (2.7)	30 (3.9)	2 (2)	
Wealth tertile	Richest	83 (42.3)	90 (35.4)	237 (31.1)	23 (23.5)	<0.001
	Middle	72 (36.7)	94 (37)	253 (33.2)	36 (36.7)	
	Poorest	41 (20.9)	70 (27.6)	273 (35.8)	39 (39.8)	
Mosquito net use	Always	138 (66.0)	202 (71.6)	441 (55.1)	49 (45.8)	<0.001
	Sometimes	12 (5.7)	15 (5.3)	57 (7.1)	7 (6.5)	
	Never	59 (28.2)	65 (23)	303 (37.8)	51 (47.7)	
Fever in the last 24 hours	Yes	12 (5.7)	10 (3.6)	82 (10.3)	19 (17.8)	<0.001
	No	199 (94.3)	271 (96.4)	716 (89.7)	88 (82.2)	

Around half (N = 678; 47.2%) of children tested positive for malaria via the RDT. After stratifying by setting, the significant association between anemia and malaria only persisted for those children living in urban neighborhoods (Table 3).

**Table 3:** Malaria and anemia by setting in children, Bata district, June-August 2013.

Anemia		RDT		p value
		Negative n (%)	Positive n (%)	
Urban	Non anemic	119 (21.5)	31 (9.8)	<0.001
	Anemic	435 (78.5)	284 (90.2)	
Rural	Non anemic	23 (12.4)	32 (9)	0.213
	Anemic	162 (87.6)	323 (91)	
Total	Non anemic	142 (19.2)	63 (9.4)	<0.001
	Anemic	597 (80.8)	607 (90.6)	

**Fig 2:**
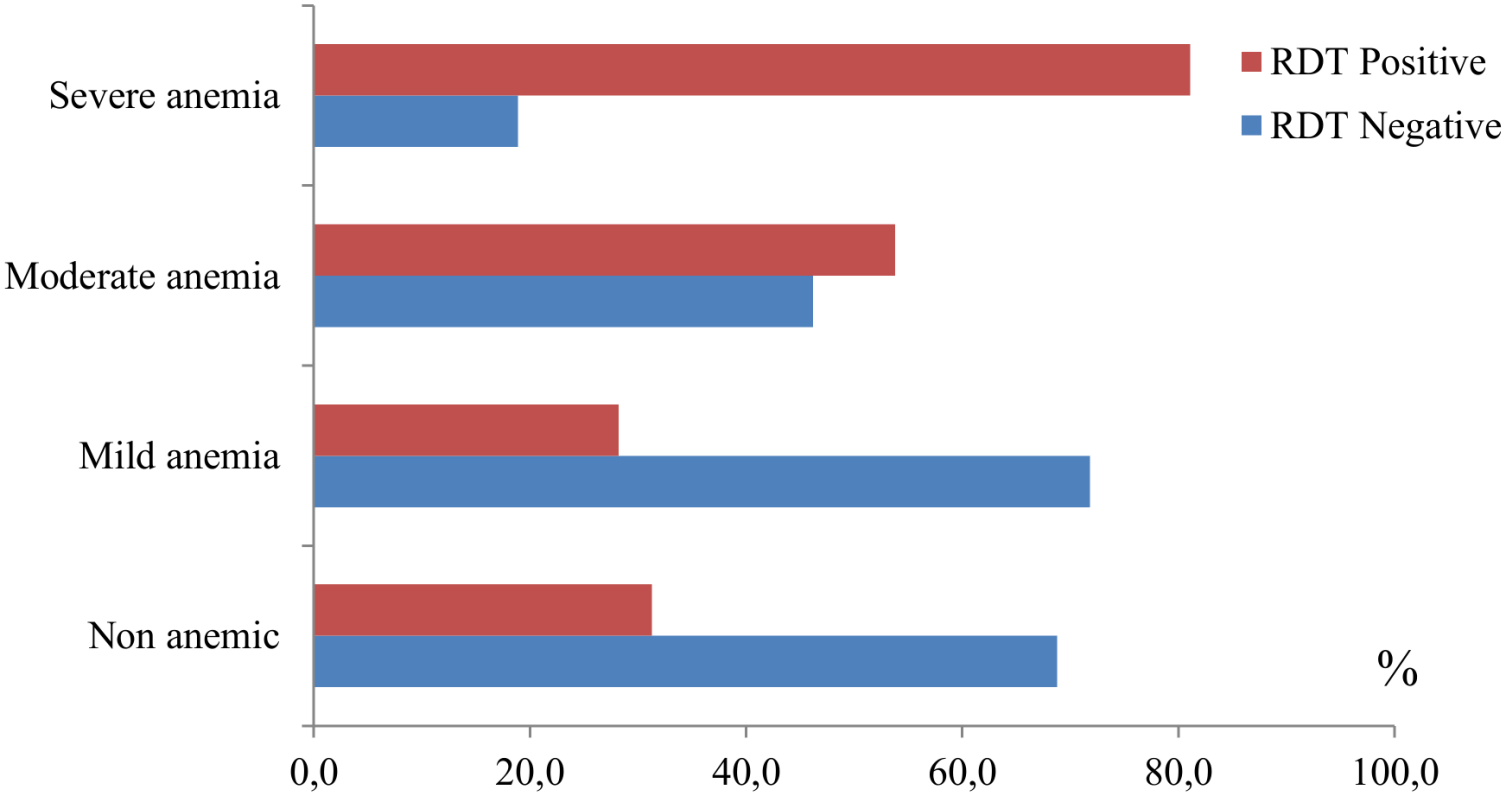
Distribution of malaria by anemia severity in children, Bata district, June-August 2013. RDT: Rapid diagnostic test.

The distribution of malaria positives by anemia severity is summarized in Fig 2. Moderate and severe anemia prevalence were higher among those children RDT positives (p<0.001), while no significant difference was found in mild anemia by malaria status.

Results from the bivariate analysis are summarized in Table 4. In urban sites, infants aged up to 5 years old were less likely to have anemia (crude prevalence ratio (cPR): 0.92; 95%CI: 0.85–0.99). Children with malaria had a higher anemia prevalence compared with non-malaric children in urban sites (cPR: 1.15; 95%CI: 1.08–1.22). Also caregiver´s educational level, family incomes´source, family wealth tertile and having fever in the last week were postitively associated with anemia in children living in urban neighborhoods, while none significant association was found between anemia and the independent variables in those children living in rural villages. Therefore, the multiple analysis was only carried out for children from urban spots.

**Table 4:** Factors associated with anemia in children aged 2 months-15 years old, Bata district, June-August 2013.

Variables		Urban			Rural		
		n° anemic (%)	Bivariate		n° anemic (%)	Bivariate	
			Prevalence Ratio	95% CI		Prevalence Ratio	95% CI
Sex	Female	372 (84.5)	1		254 (91.7)	1	
	Male	355 (80.9)	1.13	(0.95–1.35)	232 (87.5)	0.79	(0.57–1.09)
Age group	<= 12 months	234 (86.3)	1		116 (90.6)	1	
	13–59 months	264 (82.8)	0.96	(0.89–1.02)	178 (90.8)	1.00	(0.93–1.08)
	≥ 5 years	229 (79.2)	0.92	(0.85–0.99)**	192 (88.1)	0.97	(0.90–1.05)
RDT malaria	Negative	435 (78.5)	1		162 (87.6)	1	
	Positive	284 (90.2)	1.15	(1.08–1.22)**	323 (91.0)	1.04	(0.98–1.11)
Ethnic group	Fang	591 (83.0)	1		419 (89.7)	1	
	Combe	63 (85.1)	1.01	(0.95–1.08)	30 (96.8)	1.05	(0.95–1.16)
	Others	73 (78.5)	0.96	(0.88–1.05)	37 (84.1)	0.95	(0.85–1.06)
Caregiver´s educational level	No formal education/Incomplete primary school	37 (92.5)	1		36 (87.8)	1	
	Primary school	201 (82.7)	0.89	(0.80–0.99)**	208 (91.6)	1.04	(0.92–1.18)
	Secondary school	425 (81.0)	0.87	(0.79–0.96)**	188 (89.1)	1.01	(0.90–1.15)
Family incomes´source	Agriculture/fishing/Chasing	23 (92.0)	1		111 (93.3)	1	
	Employee	415 (81.7)	0.89	(0.79–1.00)*	164 (88.6)	0.95	(0.89–1.02)
	Self-employed	220 (81.8)	0.89	(0.78–1.01)*	173 (88.7)	0.95	(0.89–1.02)
	Others	31 (93.9)	1.17	(0.31–4.46)	8 (88.9)	0.95	(0.54–1.66)
Wealth tertile	Richest	289 (79.0)	1		61 (91.0)	1	
	Middle	288 (83.0)	1.05	(0.98–1.13)	96 (88.9)	0.98	(0.88–1.08)
	Poorest	101 (91.8)	1.16	(1.08–1.26)**	283 (90.4)	0.99	(0.91–1.08)
Mosquito net use	Always	520 (81.8)	1		173 (89.2)	1	
	Sometimes	32 (80.0)	0.98	(0.83–1.15)	47 (92.2)	1.03	(0.94–1.14)
	Never	164 (85.9)	1.05	(0.98–1.12)	257 (89.5)	1.00	(0.94–1.07)
Fever in the last week	No	144 (90.6)	1		119 (91.5)	1	
	Yes	572 (80.7)	1.12	(1.06–1.19)**	357 (89.3)	1.03	(0.96–1.09)

RDT: Rapid diagnostic test; OR: odds ratio;

* <0.10;

** <0.05

The difference between the age groups remained significant in the final model, showing that the prevalence of anemia was higher in urban children aged 13–59 months (adjusted PR (aPR): 0.92; 95% CI: 0.85–0.99) and 5 years and over (aPR: 0.85; 95% CI: 0.78–0.94) compared to younger children. Children with RTD positive results also had a higher prevalence of anemia (aPR: 1.19; 95% CI: 1.12–1.28). Having fever in the last week showed a significant positive association with anemia (aPR: 1.07; 95% CI: 1.01–1.15). With regard to household socio demographic characteristics, the association between the caregiver´s educational level and the prevalence of anemia lost significance because of the covariance with the family incomes´source and their wealth tertile. Belonging to the poorest wealth tertile were positively (aPR: 1.14, 95% CI: 1.05–1.24) and children’ parents being employees (aPR: 0.86, 95% CI: 0.76–0.96) or self-employes (aPR: 0.86, 95% CI: 0.76–0.97) *vs*. working in agriculture and/or fishing negatively associated with anemia among urban children (Table 5).

**Table 5:** Factors associated with anemia in children 2 months-15 years old living in urban settings, Bata district, June-August 2013.

VARIABLES		Urban		
		aPR	95% CI	p value
Age group	<= 12 months	1		
	13–59 months	0.92	0.85–0.99	0.032
	≥ 5 years	0.85	0.78–0.94	0.001
RDT malaria	Negative	1		
	Positive	1.19	1.12–1.28	<0.001
Caregiver´s educational level	No formal education/Incomplete primary school	1		
	Primary school	N.S.		
	Secondary school	N.S.		
Family incomes´source	Agriculture/fishing/chasing	1		
	Employee	0.86	0.76–0.96	0.007
	Self-employed	0.86	0.76–0.97	0.011
	Others	0.97	0.83–1.12	0.668
Wealth tertile	Richest	1		
	Middle	1.03	0.96–1.11	0.399
	Poorest	1.14	1.05–1.24	0.002
Fever in the last 24 hours	No	1		
	Yes	1.07	1.01–1.15	0.022

RDT: Rapid diagnostic test; N.S.: Not significant; aPR: adjusted prevalence ratio; CI: confidence interval

## Discussion

The prevalence of anemia among 2 months-15 years old children in Bata was 85.2%, above the WHO country estimates of 66% for the 6–59 months age in 2011 [13]. Our rate was also higher than that shown in a study carried out in continental Equatorial Guinea among children below the age of five years (69.3%) [22]. Thus, the burden of anemia remains high and a “severe public health problem” as per the WHO definition [23]. Not surprisingly, children with malaria were more prompted to be anemic, settling that children are particularly vulnerable to both the threat of malaria and anemia. Child's age and worse household conditions also contributed to the prevalence of anemia, but only in urban settings.

To our knowledge, this is the first study showing the magnitude of anemia and malaria in children aged from 2 months to 15 years old in Bata district. Both malaria infection and anemia prevalence were previously described in Bioko Island (insular Equatorial Guinea) [24]. In that study, a decrease in anemia was observed four years after high coverage, multiple malaria control interventions were introduced. Unfortunately, although similar package of interventions was introduced on the mainland in 2007, funding restrictions since 2011 considerably limited their scope in the EG continental area [25].

Severe anemia prevalence was particularly high in children aged 2–12 months and over 5 years old. The increased risk of anemia in children below the age of 12 months is consistent with findings from other countries [26,27]. This vulnerability is possibly due to the increased need for iron at this development stage of a child and the inadequate introduction of iron-rich foods after weaning [28]. Regarding school-aged children (over 5 years old), possibly children are likely starting school with untreated anemia, as nutrition interventions targeting preschoolers are very limited and inconsistent in this area [22]. Other socio-demographic (such as caregiver´s educational level, family incomes´ source wealth index) and individual factors (use of bed net) were significantly related with the grade of anemia severity. Anemia is the result of a wide variety of causes and health determinants that often coexist together [29]. Iron deficiency is the most common cause leading to anemia [2], but infectious diseases such as malaria, worm infestation and schistosomiasis [30–32], deficiencies of other essential micronutrients [5,30] and socioeconomic factors such as maternal education and low household income [12]; are also important determinants of anemia, particularly in this kind of contexts.

The prevalence of anemia and severe anemia were higher in rural than urban settings. This result is concordant with common findings which suggest the existence of worse health indicators in African rural settings [33]. Interestingly, the significant association between anemia and current malaria only persisted in urban neighbourhoods after the stratification by setting was carried out. Although the malaria effect in anemia prevalence is undeniable in endemic countries, other several factors influence the progressive fall in hemoglobin that is observed during childhood in areas of stable malaria transmission [1], such as poor nutritional status and poor sanitation, micronutrient deficiencies, intestinal helminths, HIV infection, and hemoglobinopathies [9,31,34,35]. Moreover, differences in the socio-demographic and nutritional factors associated with *P*. *falciparum* infection for rural and urban populations have also been described [36]. Probably, there is a cumulative effect of these factors in those children living in rural villages that we were not able to measure in the current study. Thus, additional risk factors that play a significant role in anemia development, such as the presence of intestinal parasitoses and micronutrient deficiencies, should be further investigated in next studies.

A number of factors significantly related to anemia were found in urban settings, but none in rural villages. Anemia in urban areas displayed a heterogeneity and complexity that differed from the rural environment, which has important implications for anemia control [37]. In the urban settings, children living in poorer households were at greater risk of having anemia than those from richer households. Previous studies have demonstrated associations between anemia and indicators of poor socioeconomic conditions in African urban areas. Ngnie-Teta *et al*. analyzed the risk of anemia in relation to the family's wealth and observed a greater risk of anemia among children who were at the lowest household living standard [38]. In Equatorial Guinea, Custodio *et al*. found that children who lived in households with lower socio-educational levels were at greater risk of anemia [22]. African countries undergoing a socio-demographic transition are particularly vulnerable to health inequities. People who migrate from rural areas generally settle in poorly constructed houses in densely populated and underdeveloped peri-urban areas, highlighting the problem of unregulated rapid urban growth on the health of the population [9,39].

## Limitations

The present study was conducted in Bata district, thus, the findings may not be generalizable to the whole country. The reader is also alerted to limitations inherent to the nature of this study, namely its descriptive nature; the direction of the relationship between malaria and anemia, given the cross-sectional design of our study, cannot be further elucidated from these data.

Overall, the prevalence of anemia identified from this survey was quite high. It cannot be denied that the use of the WHO cutoffs in this population might be responsible for an artefactually high prevalence, at least partially. However, there is no background of alternative cutoff used locally that can be employed in sensitivity analyses. Thus, further analyses including the use of alternative cutoffs are needed in the study area.

Pre-analytical bias due to the complex field logistics might have also occurred. To reduce these potential biases, detailed guidelines were prepared and piloted prior to the field work. All these protocols were also tested by internal control before and during the field work.

Anemia, micronutrient deficiencies and infectious diseases often coexist and exhibit complex interactions. Several micronutrients have immunomodulating functions and thus influence the susceptibility of a host to infectious diseases and the course and outcome of such diseases [40]. Therefore, a primary limitation of our study is that we did not obtain data on iron status; other nutritional indices and more detailed information on children´s infectious status, from a qualitative and quantitative approach (i.e. complete assessment of infectious morbidity and measurement of micronutrients). These calls for the need to undertake further investigations not only to substantiate the data obtained in the present study but also to check new hypothesis which may have emerged.

## Conclusions and recommendations

This is the first time that anemia and malaria prevalences and their co-existence have been described in children aged 2 months to 15 years in Bata district, the biggest area in continental Equatorial Guinea. In addition, difference by age group and setting were also assessed. Thus, we believe that the current manuscript implies an important advance on both diseases knowledge in EG, and we sincerely expect to contribute to their control in this area.

A very high prevalence of anemia prevalence was found in Bata district, with marked urban-rural disparities. However, no specific action has yet been implemented for combating anemia, not even the prophylactic distribution of ferrous sulphate in children, one of the most vulnerable groups. Thus, strategies targeted at fighting anemia and its main causes are clearly paramount in this country.

A review of 29 studies in Africa revealed that malaria control activities increased Hb levels by an average of 0.76 g/dL [41]. In Bata district, there were improvements in malaria control from 2007 to 2012 thanks to the EGMCI. Since then, the low accomplishment of national treatment policies and the stock shortages of first line treatment has worsened the situation [19]. Access to the malaria first-line treatment and other malaria control strategies, in conjunction with micronutrient supplementation, including iron, are needed to move forward in anemia prevention and control in EG.

The prevalence of anemia and malaria were higher in rural than in urban settings. On the other hand, anemia in urban areas displayed a heterogeneity and complexity that differed from the rural environment. Although many of Africa’s health problems are common to both urban and rural environments, the epidemiology of several infectious diseases and the challenges to prevention and control can differ [39]. The marked urban-rural variation indicates the importance of targeting specific areas or districts, with special emphasis in those worst affected. Anemia in Africa is a multifactorial process which is consequence of distinct and potentially interactive causes [42], therefore interventions aimed at anemia prevention and treatment should be coordinate with other public health actions to guarantee their optimal results.

## Supporting information

S1 Table Anemia severity by age group in children, Bata district, June-August 2013. (XLSX)
